# Neurorobotics—A Thriving Community and a Promising Pathway Toward Intelligent Cognitive Robots

**DOI:** 10.3389/fnbot.2018.00042

**Published:** 2018-07-16

**Authors:** Jeffrey L. Krichmar

**Affiliations:** ^1^Department of Cognitive Sciences, University of California Irvine, Irvine, CA, United States; ^2^Department of Computer Science, University of California, Irvine, Irvine, CA, United States

**Keywords:** brain-based devices, evolutionary robotics, embodied cognition, cognitive robotics, Neural Darwinism, neuromorphic engineering

## Abstract

Neurorobots are robots whose control has been modeled after some aspect of the brain. Since the brain is so closely coupled to the body and situated in the environment, Neurorobots can be a powerful tool for studying neural function in a holistic fashion. It may also be a means to develop autonomous systems that have some level of biological intelligence. The present article provides my perspective on this field, points out some of the landmark events, and discusses its future potential.

## Introduction

**Figure 1:**
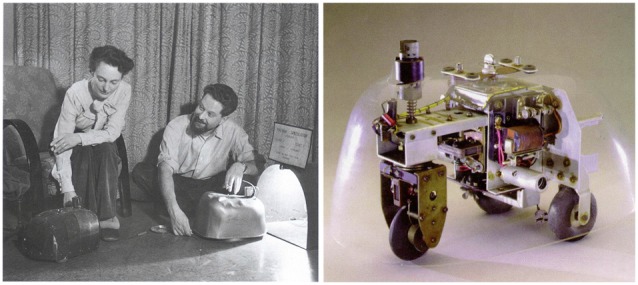
Grey Walter’s tortoises. **Left**. Picture taken from http://cyberneticzoo.com. **Right**. Photo taken from http://www.extremenxt.com.

I have been involved in Neurorobotics for over 20 years now, long before the field had a name. I thought I would take this time and space to reflect on how the field got started and where I think it is heading. Many believe Neurorobotics got its start with Grey Walter’s Tortoises^1^, which were built prior to the digital age and had rudimentary light sensors and collision detectors controlled by a simple analog circuit (Figure 1). However, these simple brains produced seemingly complex behavior that we might call intelligent.

Another seminal moment for the field was the *Vehicles* thought experiments by famed neuroanatomist Valentino Braitenberg (Braitenberg, 1986). Each chapter of this short book introduced a simple robot or vehicle that was a lesson in neuroscience. For example, by connecting the left light sensor to the right motor of these imaginary robots, and vice versa, Braitenberg described the difference between contralateral and ipsilateral connections and their effect on behavior. Using Vehicles, he introduced concepts of sensorimotor loops, inhibition and valence with these simple thought experiments.

I also would argue that the work of Rodney Brooks in the early 90s was important for the establishment of Neurorobotics. At this time, Good Old Fashion AI (GOFAI) was dominating the field of “intelligent” robots (Kuipers et al., 2017). Following GOFAI, these robots had a representative real-world model, a reasoning engine, and rule-based systems to guide the robot’s behavior. Brooks wrote two very influential articles that turned the field on its head: *Intelligence without reason* (Brooks, 1991a) and *Intelligence without representation* (Brooks, 1991b). The idea was similar to Grey Walter in that sensorimotor integration led to seemingly natural behavior. Brooks introduced the *subsumption architecture* as a means to trigger primitive behaviors and arbitrate between them. Their robots resembled insects as they scurried around avoiding obstacles, finding objects, and responding to changes in the environment. Later on, the subsumption architecture was used to create robots that moved like humans or interacted naturally with humans.

Unlike other robots at the time they were “Fast, Cheap and Out of Control”^2^. Just like biological organisms. Their work made the point that the selection and interaction of low level processes could lead to intelligent behavior. From a neuroscience point of view, this has similarities to subcortical processing of homeostatic behaviors, such as autonomic activities, hunger, body weight regulation, neuroendocrine functions, reproductive behavior, aggression and self-preservation (Parvizi and Damasio, 2001; Venkatraman et al., 2017).

This is the backdrop (circa 1997) of where my neurorobotics story begins. I was a newly minted Ph.D. trying to figure out where I wanted to go with my research. At the time, I was working with Giorgio Ascoli on the importance of dendritic morphology (Ascoli et al., 2001a,b). Giorgio is a brilliant scientist and I was a fairly skilled computer programmer. So, the combination of the two of us led to early work generating and visualizing dendritic trees. Although my Ph.D. was in Computational Neuroscience, I had a background in computer science. More specifically, I worked on real-time and embedded systems in industry before entering academia. As exciting as the field of computational neuroanatomy and neuroinformatics was, I was more interested in the behavior of organisms under natural conditions. I thought that my industry experience might be applicable to a new line of research.

Late in 1998, I saw an opening for a postdoctoral fellow position for the Keck Machine Psychology Laboratory at The Neurosciences Institute in La Jolla, California. I was intrigued by this idea and reached out to the point of contact, Olaf Sporns. After an encouraging conversation with Olaf, he suggested that I should visit The Neurosciences Institute for an interview.

**Figure 2:**
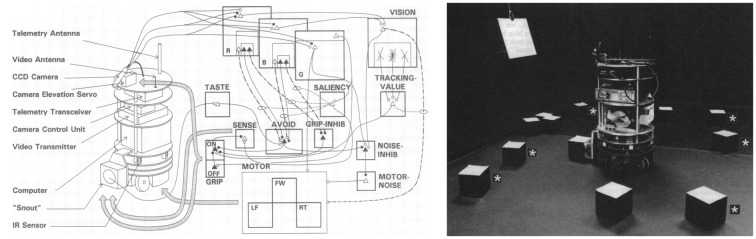
Darwin IV Brain-Based Device. **Left**. Neural network model to control Darwin IV’s behavior. **Right**. Darwin IV in a conditioning task. Adapted from Edelman et al. (1992) with permission.

The Neurosciences Institute was a unique place. The director was Nobel Laureate Gerald Edelman. In addition to his work in immunology, which led to the Nobel Prize, he introduced a theory of the nervous system called Neural Darwinism: The Theory of Neuronal Group Selection (Edelman, 1987, 1993). The theory suggested that there was selection of neural circuits during development through synaptic pruning, and selection of groups of neurons during adulthood through reentrant connections. Important for neurorobotics was the notion of value systems to tie environmental signals to neuronal groups, which led to the selection of behaviors important for survival. Because of this linkage, or as Edelman would say, “The brain is embodied, and the body is embedded in the environment,” their group developed the Darwin series of Brain-Based Devices (Reeke et al., 1990; Edelman et al., 1992). Another phrase that drove this work, was “the world is an unlabeled place,” which meant that perceptual categories must be selected through experience, rather than supervision. These Brain-Based Devices were robots^3^ with large-scale neural networks controlling their behavior (Figure 2). However, these were not the feedforward input layer→hidden layers→output layer neural networks that were popular then and became the deep neural networks of today. The Brain-Based Device’s neural networks had anatomical details that resembled biological neural networks. There were sensory streams, top-down connections, long-range connections between regions that were bi-directional, as well as local lateral excitation and inhibition within brain regions.

By the time I visited The Neurosciences Institute, they had already developed Darwin V, a Brain-Based Device with an artificial nervous system that could learn preferences and predict the value of objects (Almassy et al., 1998). This was what I dreamed of doing, but they had a 10-year head start over me, and they were like no other group at the time.

**Figure 3:**
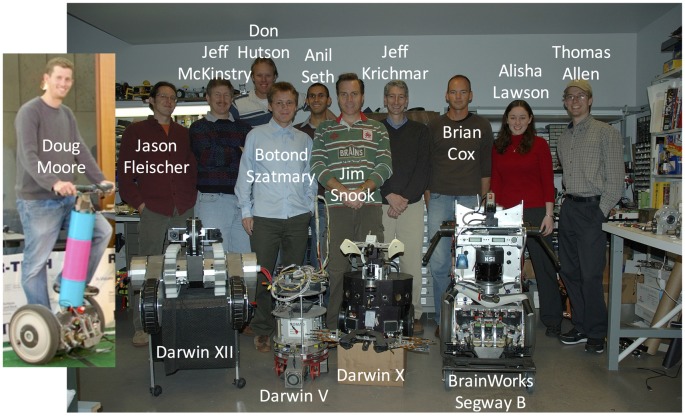
The Neurosciences Institute Build-A-Brain team (circa 2007). The team was a mix of PhD research fellows (Jason Fleischer, Jeff Krichmar, Jeff McKinstry, Anil Seth, Botond Szatmary), engineers (Brian Cox, Donald Hutson, Doug Moore), and student interns (Thomas Allen, Alisha Lawson).

My visit to The Neurosciences Institute was almost too good to be true. I bought into the overall mission of the institute that Edelman had created, and I enjoyed discussing research with Sporns and his colleagues. It didn’t hurt that La Jolla was beautiful, especially for someone visiting from the East Coast of the United States during December. But, one thing that helped seal the deal was meeting Jim Snook, their engineer on staff. Jim was a self-taught engineer who was both creative and talented. I can’t say enough how invaluable a person like this is for running a neurorobotics lab. I knew if I joined their team, I could concentrate on the science knowing that there was someone who could keep the machines running (see Figure 3). I have been extremely fortunate over the years to work with some very talented engineers, including Jim Snook, Donald Hutson, Doug Moore, Brian Cox and Liam Bucci. Which is good because over the years I broke a lot of machines!

Needless to say, I jumped at the opportunity, moved out to San Diego, and began my career in the field of Brain-Based Devices, cognitive robots and neurorobotics.

## Early Years

**Figure 4:**
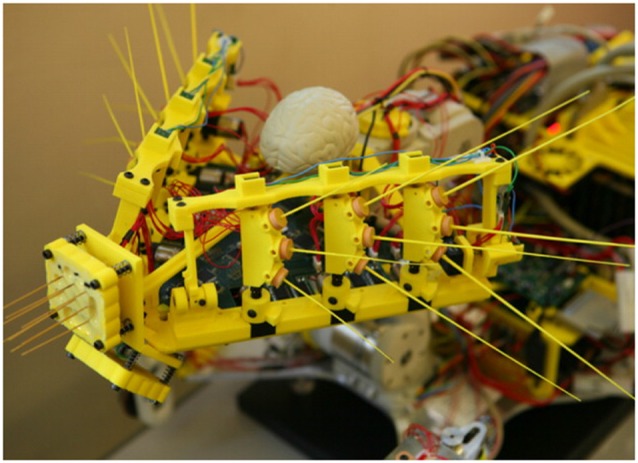
Whiskerbot from the University of Sheffield. Whiskerbot had two active whiskers and a detailed neural network model to convert whisker deflection signals into simulated spike trains. Adapted from Pearson et al. (2011) with permission.

My coming out party in this research area was the Simulation of Adaptive Behavior (SAB) conference in 2000. We reported on Darwin VII, our brain-based device that was capable of perceptual categorization (Krichmar et al., 2000). At this time, there were just a few like-minded research groups investigating how embodied computational neuroscience models could be used as a tool for understanding brain and behavior. For example, Tony Prescott and his group at the University of Sheffield was developing robotic models of action selection based on the basal ganglia (Girard et al., 2003; Prescott et al., 2006). This group was also studying whisking in the rodent, and developing a robotic sensorimotor circuit with biomimetic whiskers (Pearson et al., 2011). Figure 4 shows their Whiskerbot, which was completed around 2005.

Also related to neurorobotics was the work by Dario Floreano’s group on evolutionary robotics and Rolf Pfeifer’s group on morphological computation.

**Figure 5:**
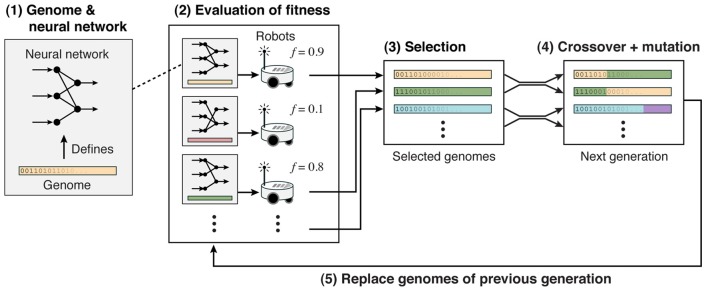
Evolutionary neural network controllers for robots. Adapted from Floreano and Keller (2010) with permission.

Nolfi and Floreano (2000) established the field of Evolutionary Robotics. They used evolutionary algorithms to evolve neural networks that supported a range of behaviors from navigating mazes to developing predator-prey strategies (Floreano and Keller, 2010). Figure 5 shows the strategy: (1) A genome defines the neural network controller, which has input neurons receiving inputs from sensors, and output neurons that control actuators. These genomes could directly define the weights or indirectly define plasticity and topology rules. (2) The fitness was based on the robot’s performance in a task. (3) The best neural network controllers were selected and (4) subject to mutation and/or crossover. (5) From this selection, a new population of neural network controllers was generated.

Pfeifer and Bongard (2006) had the insight that the “Body Shapes the Way We Think”. They suggested that biological organisms perform morphological computation, that is, certain processes are performed by the body that would otherwise be performed by the brain. By ingenious use of body plans and materials, they showed how the morphology of the robot could lead to intelligent behavior with minimal neural control. For example, their quadruped puppy had a small neural network to control gaits, but the main control of the gaits came from the springiness of its hips and knees, and the amount of friction on its feet (Hoffmann et al., 2012). I remember visiting Pfeifer’s AI lab in Zurich and talking with his students. Often their most important design consideration was choosing the proper materials. In the case of the puppy, they chose a material used to cover skis that had just the right amount of friction. The combination of springy legs and sticky feet allowed the puppy to adapt its gait over a wide variety of terrains due to the morphology’s dynamic interaction with the environment, rather than a complex control policy. The movement of the puppy moved far more naturally than other legged robots at this time.

Despite these advancements in the field, the Darwin series of automata was an outlier. However, it should be mentioned that other groups had similar goals to produce brain inspired robots and develop architectures that support this effort. For instance, the Computational Embodied Neuroscience approach (Caligiore et al., 2010), whose aim was to develop systems level models that account for an increasing number of experiments, while avoiding at the same time to build *ad hoc* models which account for only specific single experiments. Another related approach is Cognitive Developmental Robotics (Asada et al., 2009), which is a synthetic approach that developmentally constructs cognitive functions. In these approaches, and forgive me if I neglect other related approaches, the simulations are constrained by our knowledge about cognitive science, neuroscience, and psychology, and experiments are carried out on a physical embodied system situated in the real world.

Our own group followed up to the SAB perceptual categorization work by demonstrating that Darwin VII was capable of first and second order conditioning with visual and auditory stimuli (Krichmar and Edelman, 2002). The neural network that controlled its behavior was approximately 20,000 neurons and nearly 5,00,000 synaptic connections, all of which had to updated in real-time to keep up with the active vision and sensors. Invariably, when I gave talks on Darwin VII and other brain-based devices at this time, the question would come up as to why we needed so many neurons. Such behavior could be realized with a far smaller neural network. For example, work by Floreano and Keller (2010) on evolutionary robotics showed that small neural networks were sufficient to support interesting behaviors. Moreover, the dynamics of passive walkers showed that complex behavior, such as bipedal locomotion could be observed with little or no control (Collins et al., 2005).

Although the size of the neural network often depends on the problem domain, there are practical and theoretical reasons for constructing and analyzing large-scale neural networks when studying the brain using embodied models. The practical reason is that if you want to preserve neuroanatomical pathways, such as in a neurorobotic vision experiment, you will need many neurons. For example, our model of the visual cortex that allowed us to test theories of feature binding and invariant object recognition required a neuron at every camera pixel (or receptive field) for each feature (color opponency and orientation selectivity). This resulted in a large-scale neural network to encode environmental features (Seth et al., 2004b). Compare that to our neural network model that encoded tactile features with whiskers (Seth et al., 2004a). This model required an order of magnitude smaller neural network to encode environmental features.

The theoretical reason is if you want to use neurorobots to study the brain, you need to consider both the neural dynamics and the functional neuroanatomy. When I was at the Neurosciences Institute, Edelman would sometimes ask our group “If I held a gun to your head and asked you what is the most important feature of the brain, what would be your answer?” Eugene Izhikevich, who was my co-worker and colleague at the time answered the neuron (Izhikevich, 2004). My answer was always anatomy. The brain can operate over a wide-range of neural dynamics. But, if a key brain area is lesioned through stroke for example, it can render a person to a vegetative state. For neural modeling, preserving anatomical projections leads to large scale heterogeneous architectures. Having large groups of neurons with biophysical properties leads to interesting neural dynamics, as was observed in our large-scale model of the hippocampus and surrounding regions (Krichmar et al., 2005a; Fleischer et al., 2007). In this model, the complex interplay between the entorhinal cortex and hippocampal subfields resulted in the reliance of different functional pathways at different points in the robot’s learning. Both the neuronal dynamics and anatomical pathways were necessary for realistic brain responses. Although this fidelity results in highly complex networks, it does allow one to test theories of the brain and make better predictions.

Interestingly, the question of network size does not come up anymore. With the advent of neuromorphic hardware that can support brain-scale neural networks at very low power (Indiveri et al., 2011; Merolla et al., 2014), and the resurgence of deep neural networks with many hidden layers (LeCun et al., 2015), large-scale neural networks are now in vogue. It turns out that size, in the form of many layers, is necessary to solve more challenging problems. In the brain, many anatomical regions, diverse topologies, and neuron types are necessary to handle real world challenges.

## Start of a Community

Over the next several years a neurorobotics community emerged in part due to workshops and special journal issues on the topic. I was fortunate enough to participate in several of these events. In 2004, Anil Seth, Olaf Sporns and I organized a special session on “Neurorobotic Models in Neuroscience and Neuroinformatics” at the International Conference on the SAB (Seth et al., 2005). To introduce the session, we stated that a neurorobotic device has the following properties: (1) It engages in a behavioral task. (2) It is situated in a structured environment. (3) Its behavior is controlled by a simulated nervous system having a design that reflects, at some level, the brain’s architecture and dynamics. The session included Auke Ijspeert’s research on evolving neural networks for a robotic salamander (Ijspeert et al., 2005, 2007). In this research, different motor patterns (i.e., swimming or walking) emerged due to the interaction between brain and body with the specific environment (i.e., water or land). Olaf Sporns and Max Lungarella showed how embodiment can alter and improve the information processing of a neural system (Lungarella et al., 2005). Brain-inspired navigation has made many contributions to this neurorobotics by not only suggesting how head direction cells, place cells, and grid cells contribute to rodent navigation, but also by demonstrating how these systems can lead to robot navigation. In that vein, there were several articles on the topic (Arleo et al., 2004; Banquet et al., 2005; Chavarriaga et al., 2005).

**Figure 6:**
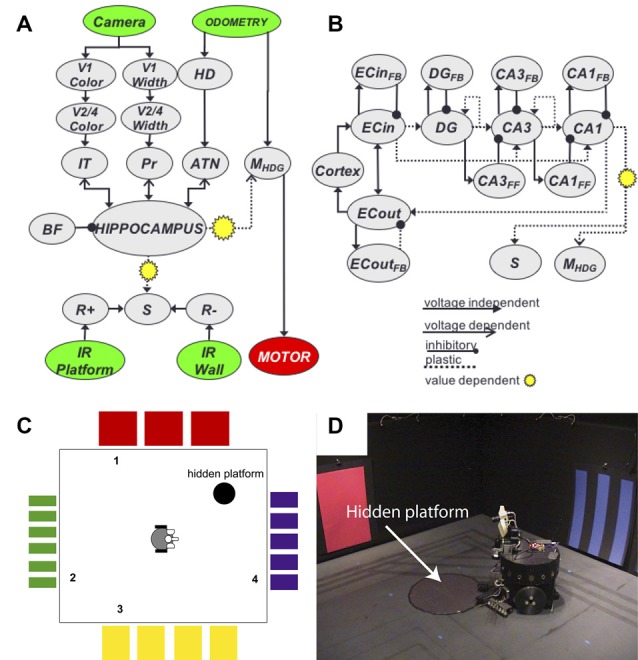
Darwin X and a hippocampal model of episodic memory. **(A)** Overall neural network architecture included neuronal groups for the visual “what” and “where” streams (V1→V2/4→IT, V1→V2/4→Pr, respectively), head direction system (HD), reward system (R+, R−, S), and hippocampus. **(B)** Subfields within the hippocampus neural group. Arrows denote synaptic projections between sub-groups. **(C)** Schematic of a dry variant of the Morris water maze. Colors denote landmarks, numbers denote starting positions of trials. **(D)** Darwin X Brain-Based Device. The hidden platform was a piece of black construction paper, which Darwin X could not see with its camera, but could detect with a downward facing IR sensor.

At this time, we introduced Darwin X, a highly detailed model of the hippocampus and surrounding areas that supported spatial and episodic memory in a Brain-Based Device^4^ (Krichmar et al., 2005a,b). Like many of these embodied navigation models, we used the robot to examine how neural activity gives rise to goal-directed behavior^5^. The robot’s task was navigating a dry variant of the Morris water maze (Figure 6). Similar to a rat, the robot was able to create routes to the hidden platform. During its experience, place cells emerged in the simulated hippocampus. What made this work special was the sheer size of the network (~100,000 neurons and 1.5 million synapses), which had to run in real-time. Because of this size and complexity, we had to develop novel methods for analyzing large-scale networks. In our case, we wanted to know what neural activity led to the firing of a place cell. In one article, we developed a method called backtracing to recursively trace back from the onset of a hippocampal place response to the sensory data that led to this response (Krichmar et al., 2005a). The other article was one of the first studies applying Granger causality to a neural network, where we analyzed what simulated entorhinal cortex, dentate gyrus, and CA3 activity led to a CA1 place cell response (Krichmar et al., 2005b). One key finding from this work was showing that the trisynaptic pathway (EC→DG→CA3→CA1) was relied on more for learning new places and routes, and that the perforant pathway (EC→CA1) was relied on more for recalling familiar places and routes. Another key finding was that this experiment demonstrated degeneracy at multiple levels. Degeneracy is the ability of elements that are structurally different to perform the same function or yield the same output, and has been shown throughout biological systems (Edelman and Gally, 2001). Darwin X showed degeneracy at the: (1) Behavioral level. No two Darwin X’s solved the maze in the same way, but they all solved the maze. We ran Darwin X through the maze protocol nine different times, with only slight differences in the connectivity of its neural network. Some Darwin X’s went directly to the platform, some bounced of walls to get to the platform. Some were perseverant, some were exploratory. (2) Neural level. We examined place cell activity on different trials where Darwin X was going through the same place on the same heading. Even under these similar conditions, a different set of neurons led to the firing of this place cell. This could only be shown in a computational model where we had access to the complete artificial brain and in a robotic system where unreliable sensing and environmental noise changes context. (3) Systems level. Darwin XI, which navigated a plus maze (Fleischer and Krichmar, 2007; Fleischer et al., 2007), received sensory input from its camera (vision), whiskers (somatosensory), compass (head direction) and laser range finder (depth/distance). Darwin XI’s spatial memory was multimodal and degenerate. Even when one or more of its sensory modalities were lesioned, Darwin XI’s behavior and place cell activity remained stable. In addition, system level tools such as Granger Causality and Dynamic Causal Modeling can reveal functional pathways in complex models (Friston, 2009). In the case of Darwin X and IX, Granger Causality showed the importance of the trisynaptic pathway when learning a novel environment, and the reliance on the perforant pathway when the environment was familiar.

Another landmark event for me was meeting Hiroaki Wagatsuma. This led to the organization of a series of workshops, articles, and discussions. Hiro coerced me into co-editing a book on the topic, which was a laborious yet rewarding experience, that eventually led to a book called, “Neuromorphic and Brain-Based Robotics” (Krichmar and Wagatsuma, 2011). This book covered a wide range of topics from neuromorphic designs, to brain architectures for robots, to philosophical considerations. There were essays on the ethics of using these robots and treating these robots as sentient entities as they become more sophisticated, as well as a chapter on using neurorobots to study consciousness.

By now, Neurorobotics was becoming more mainstream. The IEEE Robotics and Automation Magazine devoted an issue to the topic (Browne et al., 2009). There were occasionally special sessions on the topic at major IEEE robotics conferences. There were government backed consortiums devoted to studying and developing cognitive robots, such as the European Union’s iCub project (Metta et al., 2010), the Cognitive Developmental Robots initiatives in Japan (Asada et al., 2009), and the Computational Embodied Neuroscience approach (Caligiore et al., 2010). The European Union’s Human Brain Project, which is a large-scale research project for understanding the nervous system, included a Neurorobotics division headed up by Alois Knoll and Florian Rohrbein (Falotico et al., 2017). The Australian RatSLAM team was reporting results with neuro-inspired algorithms that were as good or better than state of the art localization and mapping by conventional robots (Milford et al., 2016).

**Figure 7:**
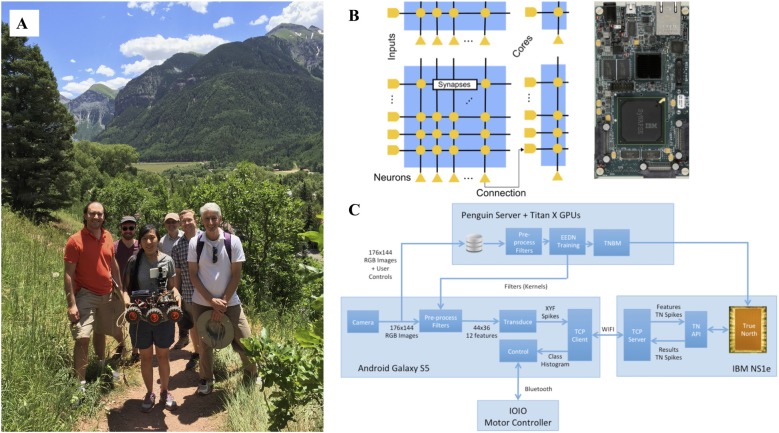
A self-driving robot using deep convolutional neural networks on IBM’s TrueNorth (TN) neuromorphic hardware. **(A)** Photograph was taken in Telluride, Colorado where the robot autonomously traversed mountain trails. From left to right are Rodrigo Alvarez-Icaza (IBM), Jacob Isbell (University of Maryland), Tiffany Hwu (University of California, Irvine), Will Browne (Victoria University of Wellington), Andrew Cassidy (IBM), and Jeff Krichmar (University of California, Irvine). Missing from the photograph is Nicolas Oros (BrainChip). **(B)** On the left, the connectivity on the IBM TN neuromorphic chip. On the right, an image of IBM TN NS1e board used in the experiments. **(C)** Data pipeline for running the self-driving robot. Training was done separately with the Eedn MatConvNet package using Titan X GPUs. During testing, a Wi-Fi connection between the Android Galaxy S5 and IBM NS1e transmitted spiking data back and forth, using the TN Runtime API. Figure adapted from Hwu et al. (2017) with permission.

Also, important around this time was the reemergence of neuromorphic engineering (Krichmar et al., 2015). Similar to the goal of neurorobotics, neuromorphic engineering was using inspiration from the brain to build devices, in this case computer architectures and sensors. Because these computers were specifically designed for spiking neural networks, algorithms that controlled neurorobots were ideal for these platforms. Our group demonstrated that a large-scale spiking neural network model of the dorsal visual stream could lead to effective obstacle avoidance and tracking on a robot (Beyeler et al., 2015). Working with IBM’s low-power TrueNorth (TN) neuromorphic chip (Esser et al., 2016), we demonstrated that a convolutional neural network could be trained to self-drive a robot on a mountain trail (Hwu et al., 2017). The robot and TN chip were all powered by a single hobby level nickel metal hydride battery (Figure 7)^6^. The circuit diagram and pipeline shown in Figure 7 can generalize to other hardware and neurorobot applications.

Because of their low-power, event-driven architectures, recent developments in neuromorphic hold great promise for neurorobot applications. In addition to our work on IBM’s chip, SpiNNaker has been used in a robot obstacle avoid and random exploration task (Stewart et al., 2016). New chips are being developed, such as Intel’s Loihi that will support embedded neuromorphic applications (Davies et al., 2018). In addition to running neural networks on specialized hardware, very low power neuromorphic vision and auditory sensors are being developed (Liu and Delbruck, 2010; Stewart et al., 2016). Similar to biology, these sensors only respond to change or salient events, and when they do respond, it is with a train of spikes. This allows seamless integration of these sensors with spiking neural networks, and their event-driven nature leads to power efficiency that’s ideal for embedded systems (i.e., robots!).

## Frontiers in Neurorobotics

A landmark event for the community was the inaugural issue of Frontiers in Neurorobotics in 2007, which was founded by Alois Knoll and Florian Rohrbein. Finally, the field had a dedicated platform to exchange ideas, and an official name. The initial year not only had articles from many of the pioneers in this field, but it also showed the breadth of the field. Tani (2007) explored top-down and bottom-up influences on sensorimotor couplings using recurrent neural networks in a humanoid robot. Angelo Cangelosi and Stefano Nolfi, who are experts in evolutionary algorithms, evolved a neural controller for reaching and grasping (Massera et al., 2007). Goodman et al. (2007) introduced their virtual neurorobotic environment, which could support very large-scale neurobiologically inspired networks. Philippe Gaussier’s group described their latest results on hippocampal inspired navigation on robots (Cuperlier et al., 2007). Finally, Steve Potter used real neurons in a multielectrode array to control a robotic arm that painted artwork (Bakkum et al., 2007).

## Future Outlook

Neurorobotics and cognitive robotics is now a vibrant, active field. Looking at some of the most recent articles in Frontiers in Neurorobotics, many of the same issues, such as motor control, navigation, mapping and developing neural networks remain. I personally would like us as a community to focus on more general cognition. Too often, present company included, we focus on a particular brain area or behavior. However, biological organisms are the ultimate multi-taskers and can readily adapt to new situations. Many of us, again present company included, preach on coupling brain, body and environment, but focus too much on the brain. The same could be said of neuroscience where currently the focus is on detailed studies of brain components and neurotechnology to gather more data. In contrast, Krakauer et al. (2017) point out that the goal of neuroscience is to understand behavior, thus we should be studying the brain in the context of naturalistic behaviors. Many roboticists focus too much on the body and simplify the robot’s behavior. Overall, the field needs to take a more holistic approach. Brains and bodies co-evolved to develop more successful behaviors in a dynamic, challenging world. However, the body often leads the brain, and its morphology is critical to what we call intelligence (Pfeifer and Bongard, 2006; Krichmar, 2012). The notion of “morphological computation” in which processes are performed by the body and its exploitation of the environment, rather than by a central control system (Pfeifer and Bongard, 2006), could greatly impact how we understand the brain, body and environment (Clark, 1996), and how we design future neurorobots. As discussed, the morphology of passive walkers relieved the necessity of complex control policies (Collins et al., 2005), and the materials used in the Whiskerbot had appropriate dynamics for recognizing objects during active whisking (Prescott et al., 2006). Although I have presented many examples of how embodied neural models have resulted in interesting behaviors in the real-world, in the future we need to develop more realistic scenarios to test our models and take into consideration how the body plan can offload brain processing.

Another reason to be optimistic about the future of this field is that now anyone can be a Neuroroboticist. Although we occasionally need to make custom robots for a particular task, most of today’s robots can be constructed from kits, off-the-shelf parts and 3D printing for a fraction of the cost when I first entered this field. For example, Nicolas Oros, who was a postdoctoral scholar in our lab, constructed a low cost, yet highly capable robot with hobby-grade platforms and Android smartphones as the computing and sensing engine (Oros and Krichmar, 2013). We have used this Android based robot idea for a wide range of research and student projects. Similar to the days of Radio Shack, there is now an online hobbyist community that makes it easy to obtain all the components necessary to build sophisticated robots. Also, open source software has made it easy to get started on programming neural networks, controlling physical robots (e.g., Robotic Operating System^7^), and creating environments for virtual robots^8^ These advances make it easy for any researcher, student, or hobbyist to get started on a neurorobotics project.

In general, this is an exciting time in Artificial Intelligence and Artificial Neural Networks. We are seeing artificial systems show better than human performance in certain tasks (Mnih et al., 2015; Silver et al., 2016). In addition, deep neural networks have been used for robotic applications with promising results. For example, an incremental deep model that extends Restricted Boltzmann Machines was developed to recognize the context of scenes (e.g., objects typically found in an office, kitchen, restroom) so that the robot can respond appropriately (Dogan et al., 2017). In another example, a Deep Belief Neural Network was trained for object recognition and robot grasping (Hossain and Capi, 2016). The DBNN was able to recognize objects in different positions and orientations by extracting object features, and then use this information to grasp objects in real time.

However, I believe there are limitations with this current, popular approach. It works in a limited domain, often requires lengthy, specific training, and may not be able to address many of the behaviors that we take for granted, but attribute to intelligence (Larson, 2017). To address these limitations Jeff Hawkins recently argued in IEEE Spectrum that intelligent systems must incorporate three key features of the brain (Hawkins, 2017): (1) Learning by rewiring; we learn quickly, incrementally, and over a lifetime. (2) Sparse representations; biological systems are under extreme metabolic constraints and need to represent information efficiently. (3) Embodiment; sensorimotor integration is observed throughout an intelligent system. I would add (4) Value systems; extracting saliency from the environment and responding appropriately (Friston et al., 1994; Krichmar, 2008), and (5) Prediction; using past experience to be more successful in the future (Clark, 2013). In the area of value systems, models of neuromodulation have been used to simulate value prediction and drive action selection (Sporns and Alexander, 2002; Cox and Krichmar, 2009; Vargas et al., 2009; Krichmar, 2013; Navarro-Guerrero et al., 2017). Predictive coding strategies using hierarchical Bayesian systems and recurrent neural networks have been used for robots to develop internal models that predict movement of object and of other robots (Park et al., 2012; Murata et al., 2017). However, future neurorobot applications will need to address all five of the above features in a holistic manner and demonstrate that the robot’s behavior can generalize across multiple task domains and over longer timeframes. I am a firm believer that neurorobotics is the ideal methodology to address these issues and limitations.

I argue that in order to get a truly cognitive system one must study and be inspired by the brain and body of natural systems. Sometimes these discussions get heated. There are those that do not feel this is a necessary requirement. However, biological intelligence is an existence proof and currently our only working model. Following its path by using Neurorobots will ultimately lead to intelligent cognitive robots and assistants.

## Author Contributions

The author confirms being the sole contributor of this work and approved it for publication.

## Conflict of Interest Statement

The author declares that the research was conducted in the absence of any commercial or financial relationships that could be construed as a potential conflict of interest.

## Footnotes

^1^For a truly delightful video of these tortoises in action, see: https://www.youtube.com/watch?v=lLULRlmXkKo

^2^“Fast, Cheap & Out of Control,” a film by Errol Morris. Distributed by Sony Pictures Classics, 1997.

^3^Because Brain-Based Devices were adaptive and stochastic, we were not allowed to call them robots, which had more fixed behavior. At least that was the idea. It sometimes caused confusion and even derision.

^4^OK. It’s a robot. Old habits die hard.

^5^As much as I think the hippocampus and spatial navigation is interesting, I do believe it’s over studied and there are many interesting, untapped brain regions and behaviors that need study. However, like the quote in Godfather III, “Just when I thought I was out, they (my students, postdocs, and collaborators), pull me back in.”

^6^So, it violated my rule of the importance of neuroanatomy. Call me a hypocrite. Still, it was a first for TrueNorth and one of the few demonstrations of a neuromorphic chip embedded in a closed loop system. Moreover, it was pretty cool to see IBM’s multi-million dollar piece of hardware cruising down a mountain trail, attached on the back of our robot with Velcro and connected to the robot’s power source with a jumper cable.

^7^http://www.ros.org/

^8^https://neurorobotics.net/
